# Advanced characterization of surface-modified nanoparticles and nanofilled antibacterial dental adhesive resins

**DOI:** 10.1038/s41598-020-66819-8

**Published:** 2020-06-17

**Authors:** Fernando Luis Esteban Florez, Artem A. Trofimov, Anton Ievlev, Shuo Qian, Adam Justin Rondinone, Sharukh Soli Khajotia

**Affiliations:** 1 The University of Oklahoma Health Sciences Center, Department of Restorative Sciences, Division of Dental Biomaterials, College of Dentistry, 1201 N. Stonewall Avenue, Oklahoma City, Oklahoma 73117 USA; 2 Oak Ridge National Laboratory, Center for Nanophase Materials Sciences, Oak Ridge, Tennessee 37831 USA; 3 Oak Ridge National Laboratory, Neutron Scattering Division, Oak Ridge, Tennessee 37831 USA

**Keywords:** Dentistry, Dental materials, Dental biomaterials, Health care, Materials science

## Abstract

Nanotechnology can improve the performance of dental polymers. The objective of this study was to modify the surfaces of nanoparticles with silanes and proteins, characterize nanoparticles’ agglomeration levels and interfaces between nanoparticles and the polymeric matrix. Undoped (n-TiO_2_), nitrogen-doped (N_TiO_2_) and nitrogen-fluorine co-doped titanium dioxide nanoparticles (NF_TiO_2_) were synthesized and subjected to surface modification procedures in preparation for Small-Angle X-Ray Scattering (SAXS) and Small-Angle Neutron Scattering (SANS) characterizations. Experimental adhesives were manually synthesized by incorporating 20% (v/v) of n-TiO_2_, N_TiO_2_ or NF_TiO_2_ (as-synthesized or surface-modified) into OptiBond Solo Plus (OPTB). Specimens (n = 15/group; d = 6.0 mm, t = 0.5 mm) of OPTB and experimental adhesives were characterized using Time-of-Flight Secondary Ion Mass Spectroscopy (ToF-SIMS), 2-D ToF-SIMS chemical imaging and SANS. SAXS results indicated that surface-modified nanoparticles displayed higher scattering intensities in a particle-size dependent manner. ToF-SIMS results demonstrated that nanoparticles’ incorporation did not adversely impact the parental polymer. 2-D ToF-SIMS chemical imaging demonstrated the distribution of Ti^+^ and confirmed nitrogen-doping levels. SANS results confirmed nanoparticles’ functionalization and revealed the interfaces between nanoparticles and the polymer matrix. Metaloxide nanoparticles were successfully fabricated, incorporated and covalently functionalized in a commercial dental adhesive resin, thereby supporting the utilization of nanotechnology in dentistry.

## Introduction

The placement of polymer-based adhesive restorations is one of the most prevalent medical interventions in the human body with more than five hundred million composite restorations placed every year^1^. Resin composite restorations became the first treatment option amongst patients and clinicians around the world due to their outstanding esthetic properties, mercury-free compositions and ultraconservative restorative techniques^2^. Despite their high acceptability and widespread use, these materials have been correlated with significant clinical shortcomings including postoperative sensitivity, shorter service lives (5.7 years) and higher incidences of failure when compared to dental amalgams. The reduced longevity observed has been attributed to a combination of factors including polymerization shrinkage, incomplete enveloping of the dentin matrix and biodegradation. This problem is exacerbated on resin composites and dental adhesive resins, because these materials were demonstrated to upregulate the aggregation and growth of oral microorganisms, and biofilms accumulated, are typically more cariogenic in nature^3^.

Furthermore, it has been suggested that the interface between synthetic and biological materials plays a vital role in shifting the microbial ecology from a state of health into a disease-associated state^4^. This shift leads to chronic chemical and biological degradation of the tooth-adhesive-resin composite interface and, ultimately, to secondary caries^5^. The occurrence of this biofilm-related disease at the adhesive-tooth interface has consistently been the primary mechanism for failure and replacement of resin composite restorations^6^. It is estimated that a total of $ 298 billion are spent globally every year for the replacement of failed restorations, which is a heavy economic burden for patients and governments, and represents an average of 4.6% of the total global health-care related expenditures^7^.

Several groups have tried to increase the service lives of bonded restorations by adding inhibitors of matrix metalloproteinases (zinc-dependent endopeptidases, MMP), antibacterial agents and monomers to current polymer compositions^8^. Experimental materials containing quaternary ammonium compounds (QAC) or quaternary ammonium dimethacrylates (QAM) were previously shown to display promising functionalities (*in vitro* and *in vivo*) against a broad variety of oral microorganisms and MMP^9–11^. A recent systematic review of the literature^12^ has indicated that the incorporation of QAMs may impact the structure of the polymeric network, degree of conversion, solvent sorption (e.g., water, ethanol and artificial saliva), polymerization shrinkage and may significantly change the biocompatibility of experimental materials against fibroblast cells^12^. Another study has demonstrated that saliva adversely impacts the antibacterial activity of QAC-containing materials due to electrostatic interactions between salivary proteins and QAC^13^.

Approaches to improve the antibacterial functionalities of dental polymers include the utilization of functionalized quaternary ammonium polyethyleneimine nanoparticles (QPEI)^14^. Even though such approach was demonstrated to result in promising initial antibacterial properties against *Streptococcus mutans*, the excess iodine attached to these highly cross-linked silica-based nanoparticles, was shown to adversely impact free-radical polymerization reactions, which inevitably led to experimental materials with low degree of polymerization, reduced mechanical properties and leaching of uncured monomers^15^. Another study has demonstrated that pyrogenic silica nanofillers undergo hydrolytic degradation within the hybrid layer independently of the level of silane functionalization^16^. Nanofillers’ dissolution within the hybrid layer was shown to result in the formation of water channels, higher water uptake, leaching of unbound hydrophilic components, and to further accelerate hybrid layer’s hydrolytic degradation^16^.

The UV-driven photocatalysis of titanium dioxide nanoparticles (n-TiO_2_) was previously shown to be effective against microorganisms relevant to public health such as bacteria (Gram-positive and Gram-negative) and viruses^17^. However, the energy dose required to achieve adequate sterilization are at levels extremely dangerous to human cells and tissues^18^, which significantly restricts the utilization of this technology in dentistry. Several studies^19–25^ have demonstrated the feasibility of shifting the n-TiO_2_ absorption behavior from the UV (200–390 nm) into the visible range (400–700 nm) of the electromagnetic spectrum by doping n-TiO_2_ with a variety of atoms including nitrogen, fluorine, copper, silver, platinum and palladium. More recently, Esteban Florez *et al*.^26^ investigated the antibacterial efficacy of highly photoactive N_TiO_2_ (size distribution 6–15 nm) synthesized by robust solvothermal reactions^27,28^. The results reported^26^ have indicated that experimental dental adhesive resins containing varying concentrations of N_TiO_2_ (50%, 67% or 80% [v/v]) displayed superior antibacterial properties against *S. mutans* biofilms (3 and 24 hours) when compared to the parental polymer in both light-irradiated and dark conditions. Despite these promising results, it is well known that the incorporation of non-functionalized nanoparticles into polymers leads to the attainment of experimental materials with inferior surface, mechanical and biological properties (germicidal, bioactivity and biocompatibility)^29^.

Consequently, surface-modification and covalent functionalization of nanoparticles is required to fabricate the state-of-the-art nanostructured composites with specific architectures, functionalities and superior mechanical, surface, chemical, physical and biological properties. Therefore, the objective of the present study was to synthesize, surface-modify, functionalize and comprehensively characterize undoped (n-TiO_2_), doped (nitrogen, N_TiO_2_) and co-doped (nitrogen and fluorine [NF_TiO_2_]) titanium dioxide nanoparticles, as well as, unaltered or experimental dental adhesive resins modified by the incorporation of 20% (v/v) of the metaloxide nanoparticles synthesized.

## Materials and Methods

### Synthesis of nanoparticles

The detailed description of the synthesis of n-TiO_2_ or N_TiO_2_ used in the present study has been reported previously in a recent publication from our laboratory^26^. Nanoparticles were synthesized (at the Center for Nanophase Materials Sciences; CNMS) in two steps using very controllable solvothermal reactions^27,28^. In the first step a solution of 1.7 g of Ti(OBu)_4_ (Aldrich, 97%), 4.6 g C_2_H_5_OH (Decon Labs, 200 proof), 6.8 g C_18_H_35_NH_2_ (Aldrich, 70%), 7.1 g C_18_H_34_O_2_ (Aldrich, 90%) was prepared and then mixed with an ethanol-water solution (4%, 18-Milli-Q; total volume = 20 mL/aliquot). Solutions prepared were transparent before mixing, however, the final solution clouded instantaneously after mixing due to hydrolysis and some micelle formation. Aliquots (20 mL/each) of the final solution were individually placed into separate high-pressure reaction vessels (Teflon-lined; Paar Series 5000, Multiple Reactor System), reacted (180 °C, 24 hours) and stirred via external magnetic field (280 rpm). Room-temperature solutions were then decanted and washed (3×, ethanol 200 proof, Decon Labs) to render pure n-TiO_2_. A portion of n-TiO_2_ in ethanol were then reacted (at 140 °C, 12 hours) with an equal volume of triethylamine (Sigma-Aldrich, 99.5%). The now nitrogen-doped titanium dioxide nanoparticles (N_TiO_2_) was then washed 3 additional times with ethanol, and the concentration of particles was gravimetrically determined to be approximately 40 mg/mL. Co-doped nanoparticles (NF_TiO_2_) were obtained in a single reaction based on step 1 with the inclusion of 5% (wt./wt.; based on Ti content) of fluorine using crystalline Ammonium Fluoride (ACS, 98%, Alfa Aesar) as the dopant source. Aliquots (10 mL/group) of the as-synthesized nanoparticles were re-suspended in deuterium oxide (D_2_O, 99.9 atom %, Sigma-Aldrich) in preparation for small-angle X-ray and neutron scattering experiments.

### Surface modification of nanoparticles

As-synthesized nanoparticles (n-TiO_2_, N_TiO_2,_ or NF_TiO_2_; ≅ 40 mg/mL) suspended in ethanol (20 mL/each) were washed (ultrapure water, 18-Milli-Q, 3 washes, 1 min/wash; 25 °C), centrifuged (8,000 rpm; 3 cycles of 15 min/each) and suspended in a pre-heated sodium hydroxide solution (NaOH, 60 °C, 15 M). Ionic solutions containing the nanoparticles were then incubated (30 min) in an orbital shaker (100 rpm) at room-temperature. Aliquots (10 mL) of NaOH-modified nanoparticles were then centrifuged (8,000 rpm; 3 cycles of 15 min/each) and re-suspended in 20 mL of (3-Aminopropyl) triethoxysilane (APTES; 85.5 mM, Sigma-Aldrich, 99%) at 90 °C for 3 hours (static conditions). Nanoparticles that were surface-modified by NAOH + APTES were then washed and centrifuged as previously described. Silanized nanoparticles were re-suspended in a buffered aqueous solution of human serum albumin (Alb; 10 mg/mL, Sigma-Aldrich, ≥99%,10% buffer) at room-temperature for 24 hours (100 rpm). Surface-modified nanoparticles (either by NaOH, APTES or Alb; or a combination thereof) were denoted as Dn-TiO_2_, DN_TiO_2_ or DNF_TiO_2_ (where D stands for any type of surface derivatization).

### Small-angle X-ray scattering (SAXS)

Aliquots (10 mL) of the as-synthesized (N_TiO_2_) or surface-modified (DN_TiO_2_) nanoparticles were re-suspended in deuterium oxide (D_2_O, 99.9 atom %, Sigma-Aldrich) containing either NaCl (0.1 M or 1.0 M) or HCl (0.1 M). Aliquots (1.0 mL) of each nanoparticle investigated (either as-synthesized or surface-modified) were then individually placed into separate wells of a disposable plastic sample holder. The SAXS experiment was then performed (8 hours irradiation/sample; 3 samples/group) on a Rigaku BioSAXS-2000 system with a rotating anode, producing CuKα X-ray radiation at 1.54 Å. SAXS, data was averaged and reduced using Rigaku SAXSlab data collection and processing software (V4.0.2 Rigaku Americas Corporation).

### Dental adhesive resins and specimen fabrication

Experimental dental adhesive resins were synthesized by manually dispersing 20% (v/v) of as-synthesized (n-TiO_2_, N_TiO_2_ or NF_TiO_2_) or surface-modified (Dn-TiO_2_, DN_TiO_2_ or DNF_TiO_2_) nanoparticles (in ethanol) into OptiBond Solo Plus (Kerr Corp.; OPTB; Composition: Self-etch primer - HFGA-GMA, GPDM, ethanol, water, MEHQ, ODMAB, CQ SE primer: 1.9; Light-cured Adhesive -Bis-GMA, HEMA, GDMA, GPDM, ethanol, CQ, ODMAB, BHT, filler (fumed SiO2, barium aluminoborosilicate, Na2SiF6), coupling factor A174 [approximately 15 wt% filled]). Disk shaped specimens (n = 15/group; diameter = 6.0 mm, thickness = 0.5 mm) of OPTB or experimental dental adhesive resins (OPTB + 20% [v/v] of either n-TiO_2_, N_TiO_2_, NF_TiO_2_ or Dn-TiO_2_, DN_TiO_2_, DNF_TiO_2_) were fabricated by individually pouring uncured materials into the separate wells of a custom-made metallic mold. Specimens were then light-cured with blue light (VALO LED, Ultradent Products, Inc., U.S.A.) from the top (1,000 mW/cm^2^ 60 s/each) following a protocol previously reported^26^.

### Helium ion microscopy (HIM)

A helium ion microscope (Zeiss Orion Nanofab) was utilized for the secondary electron imaging of specimens. Helium ion microscopy (HIM), enabled by a gas field ion source (GFIS), is a powerful imaging and nanofabrication technique compatible with many applications in materials science^30–32^. HIM offers small interaction volume of He and Ne (the two gases offered), small beam spot size, and a moderate sputtering rate^33,34^. Generally, helium allows higher resolution work, whereas neon offers milling opportunities. Additionally, the HIM can provide sharp, well resolved images from electrically insulating samples (soft, polymeric, and biological materials) without a conductive coating due to its charge compensation capabilities^32,35,36^. In the present study, specimens of each dental adhesive resin investigated were loaded into the vacuum chamber of the HIM at a pressure of ca. 2.5 × 10^−7^ Torr, and GFIS gun pressure was ca. 2 × 10^−6^ Torr. HIM imaging was performed using a focused He^+^ beam with an extraction voltage of 34 kV and acceleration voltage of 25 kV over a range of fields of view (FOV; 2 μm^2^–100 μm^2^). The beam current for imaging was measured as ca. 1.65 pA at a beam spot size of 4 μm and a 5 μm gold aperture. Imaging was done for 200 μs per pixel dwell time over 1,024 × 1,024 pixels.

### Time-of-flight secondary ion mass spectrometry (ToF-SIMS)

Time-of-flight secondary ion mass spectrometry measurements were carried out using TOF.SIMS.5-NSC instrument (ION-TOF Gmb, Germany) and allowed the surface chemistry characterization of investigated specimens fabricated with unaltered or experimental dental adhesive resins. In ToF-SIMS primary ion beam of Bi_3_^+^ clusters with energy of 30 keV, current 30 nA and beam size ~5 μm was used to extract analyte ions from the surface of each specimen. Secondary ions were further accelerated in uniform electric field and moved to the detector. Their time-of-flight was measured and allowed the calculation of mass-to-charge ratios (*m/z*) and the plotting of full mass spectra. This way ToF-SIMS allowed 2-dimmensional chemical imaging of the surface chemistry with mass resolution m/Δm = 5,000–10,000 and spatial resolution ~5 μm.

### Small-angle neutron scattering (SANS)

Nanoparticles (as-synthesized or surface-modified) or specimens fabricated with dental adhesive resins (unaltered or experimental) were individually placed inside of customized titanium cells. Each titanium cell (containing nanoparticles suspended in D_2_O or dry specimens) had two quartz windows to allow the transmission of neutrons through the specimens or samples investigated. These titanium cells were then individually mounted onto a custom-made and computer-controlled holder (capacity = 8 cells/experiment) that allowed the continuous rotation (20 rpm) of individual cells during SANS measurements. The rotation prevented nanoparticles from settling down in suspension. The SANS experiment was performed (3 hours/sample or specimen) at the Bio-SANS instrument of the High-Flux Isotope Reactor at Oak Ridge National Laboratory, following a protocol previously described^37^. The sample-to-detector distance was set to 15.5 m (main detector) and 1.13 m (wing detector) at a wavelength of 6 Å with the wavelength spread ∆λ/λ ~ 0.15. The available *q* range was 0.003 < q < 0.8 Å^−1^, where *q* = ((4π sinθ)⁄λ), and 2θ as the scattering angle. A sample aperture of 12.0 mm diameter was used for providing a sufficient neutron scattering intensity. Raw SANS data were corrected for sample transmission and background radiation by facility supplied reduction software. SANS measurements were taken at room temperature. Data analysis was performed in SASView software (National Science Foundation, DANSE project). A generalized Guinier-Porod function (GPF) was used to fit experimental data of dental adhesive resins (unaltered or experimental) containing 20% (v/v) of nanoparticles (as-synthesized or surface-modified) according to Eq. 1 by Hammouda^38^.

1 $$I(Q)=\frac{G\,}{{Q}^{s}}\exp \left(\frac{-{Q}^{2}{R}_{g}^{2}}{3-s}\right)$$

where (*G*) is a scaling factor, (*Rg*) is the radius of gyration and (*s*) is a parameter used to model three-dimensional globular objects (e.g., spheres or nanoparticles investigated).

## Results

**Figure 1:**
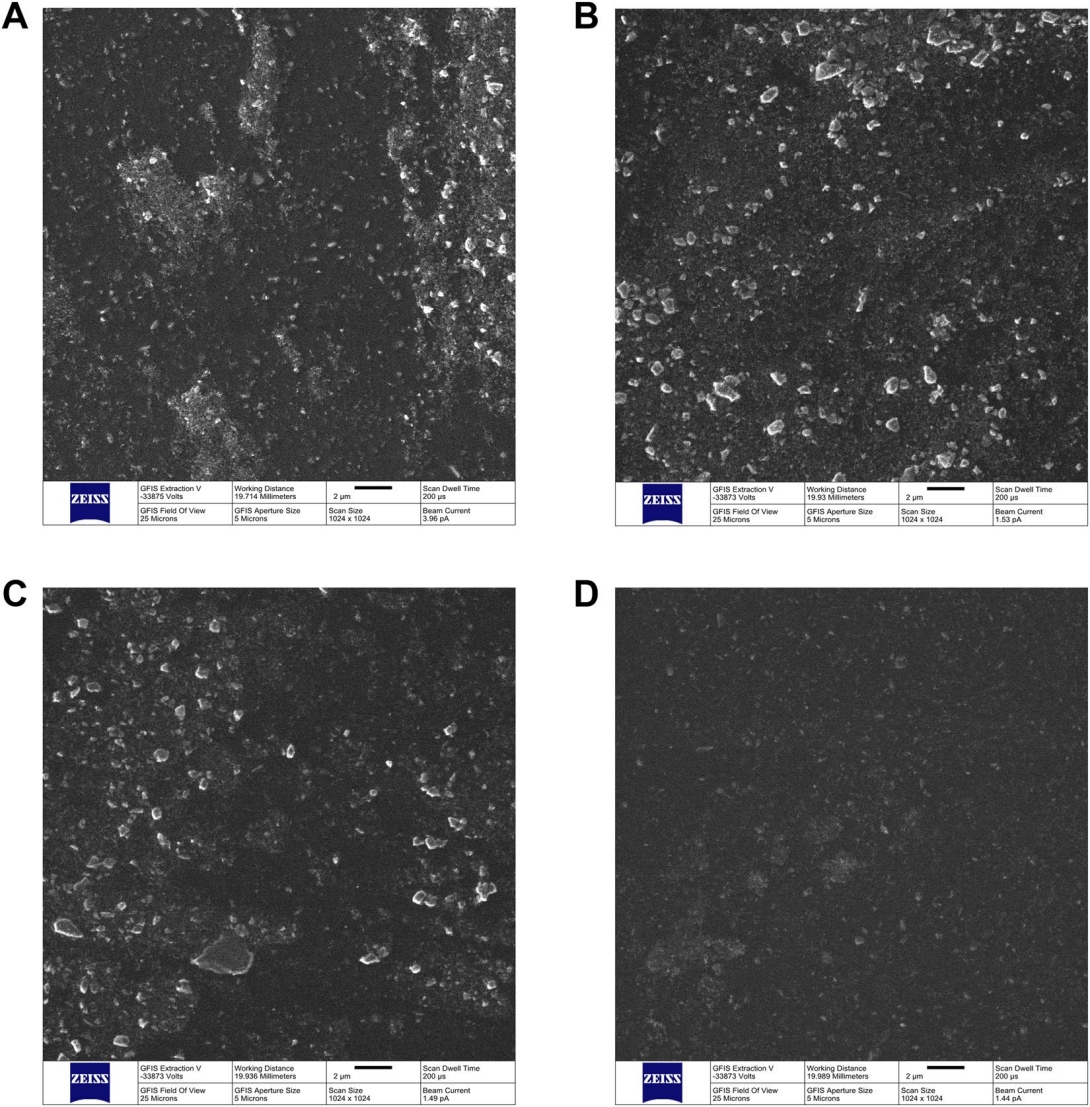
Helium-Ion Microscopy. Images (25 μm field of view) of unaltered (**A**) and experimental dental adhesive resins containing 20% (v/v) of (**B**) n-TiO_2_, (**C**) N_TiO_2_ and (**D**) NF_TiO_2_.

Figure 1(A–D) shows the surface characterization results using HIM (field of view = 25 μm^2^) of unaltered (1 A, OPTB) and experimental dental adhesive resins containing 20% (v/v) of Dn-TiO_2_ (1B), DN_TiO_2_ (1 C) or DNF_TiO_2_ (1D), respectively. Experimental adhesives were demonstrated to display topographical features that were comparable to those of OPTB, and phase separation (between nanoparticles and polymer) could not be observed (at the surface level) for all groups investigated. These findings suggest that nanoparticles (as-synthesized or surface-modified) were successfully incorporated and functionalized in the organic matrix of OPTB. In addition, it is possible to observe that surfaces investigated were dominated by the presence of micron-sized particles. This finding can be fully explained by the composition of OPTB, where salinized silica particles are used as fillers to improve the mechanical properties of OPTB.

**Figure 2:**
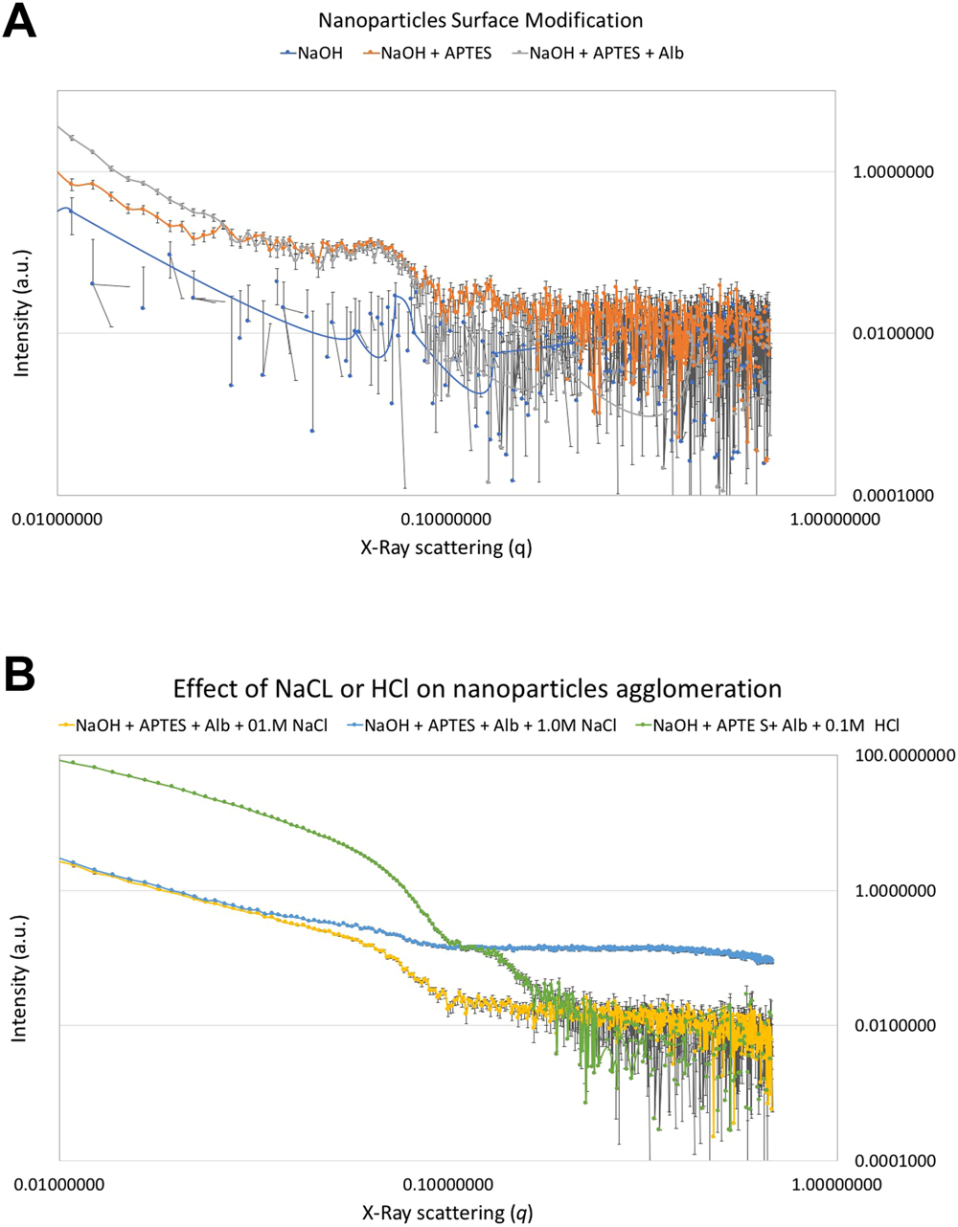
Results from the small-angle X-ray spectroscopy of surface-modified nanoparticles suspended on D_2_O. (**A**) shows the effect of surface modification (either NaOH, NaOH+APTES or NaOH+APTES + Alb) and (**B**) the effect of ionic solutions (either NaCl [0.1 M] or HCl [0.1 M]) on nanoparticles’ agglomeration levels.

Figure 2(A,B) illustrate the SAXS results for surface-modified N_TiO_2_ suspended in deuterium oxide (D_2_O) or D_2_O containing NaCl (0.1 M or 1.0 M) or HCl (0.1 M). Figure 2A clearly shows that for small values of *q* (between 0.01 and 0.1 Å^−1^), X-ray scattering intensities of surface-modified N_TiO_2_ varied in a particle-size dependent manner where NaOH+APTES + Alb > NaOH+APTES > NaOH. For larger values of *q* (between 0.1 and 1.0 Å^−1^), X-Ray scatterings indicated that surface-modified nanoparticles tend to agglomerate more when compared to as-synthesized nanoparticles. Figure 2B illustrates the impact of the utilization of deuterated ionic solutions (NaCl or HCl) on the agglomeration behavior of surface-modified nanoparticles, where it can be observed that surface-modified nanoparticles suspended in acidic media (HCl 0.1 M) displayed the best isotropic X-ray scattering behavior amongst all experimental groups investigated (green curve). These findings indicate that acidic deuterated solutions displayed large quantities of discrete particles (individually distributed) and small-sized agglomerates (15–45 nm in diameter).

**Figure 3:**
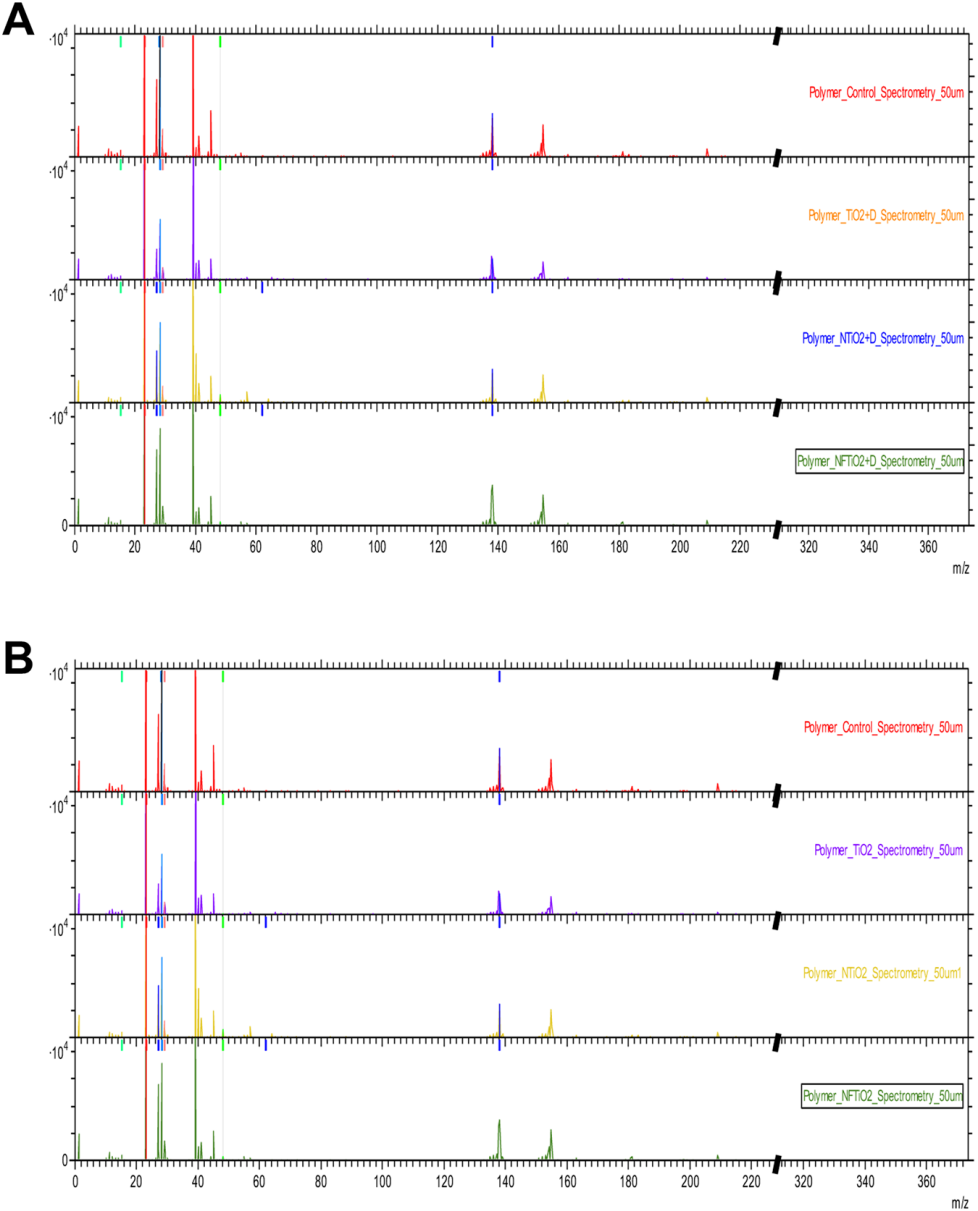
ToF-SIMS of unaltered (OPTB) and experimental dental adhesive resins containing 20% of either n-TiO_2_, N_TiO_2_ or NF_TiO_2_ (**A**) as-synthesized or (**B**) surface-modified by NaOH + APTES + Alb. From top to bottom in (**A**) and (**B**) OPTB, OPTB + n-TiO_2_, OPTB + N_TiO_2_ and OPTB + NF_TiO_2_.

**Figure 4:**
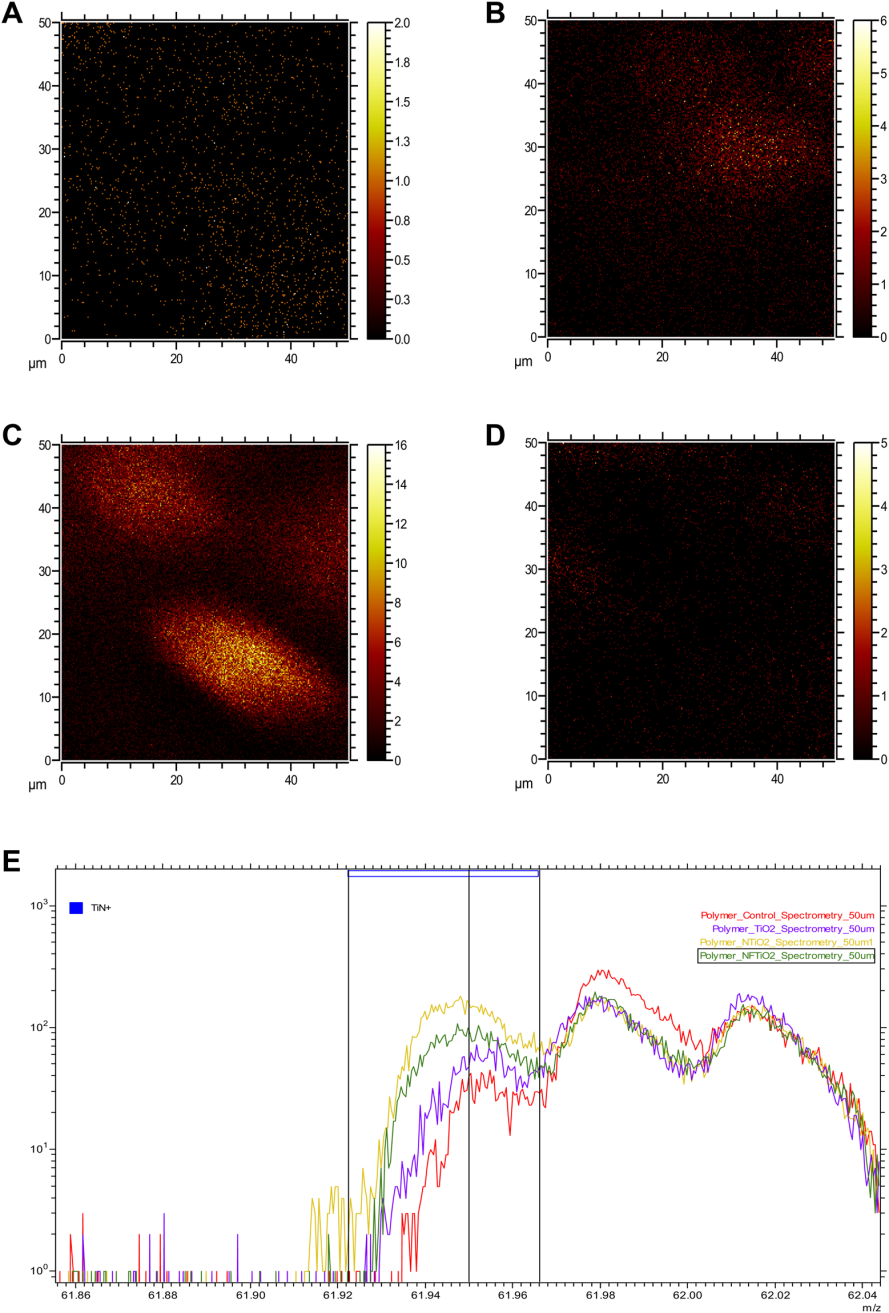
ToF-SIMS chemical imaging for Ti^+^ immobilized in (**A**) OPTB, (**B**) OPTB + n-TiO_2_, (**C**) OPTB + N_TiO_2_ and (**D**) N_F_TiO_2_. Mass spectrum analysis for TiN^+^ is shown in (**E**).

Figure 3 illustrates the ToF-SIMS results of OPTB and experimental adhesives containing 20% (v/v) of as-synthesized and surface-modified nanoparticles, where experimental adhesives displayed mass spectra that were comparable to that of OPTB, thereby supporting that the incorporation of nanoparticles investigated did not adversely impacted the organic matrix of OPTB. Figure 4(A–E) illustrates the results of the 2-D ToF-SIMS chemical imaging (FOV = 50 μm^2^) denoting the distribution of titanium (Ti^+^) within OPTB (A) and experimental adhesives containing 20% (v/v) of n-TiO_2_ (B), N_TiO_2_ (C) or NF_TiO_2_ (D). It can be observed (Fig. 4C) that specimens containing 20% (v/v) of N_TiO_2_ displayed the highest concentrations of Ti^+^. The results shown in Fig. 4E (for m/z between 61.86 and 62.04) not only confirm the findings from the 2-D chemical mapping, but also represents the first instance in dentistry, in which nitrogen-doping is mapped within the crystal lattice of titanium dioxide nanoparticles while immobilized in a commercial adhesive resin.

**Figure 5:**
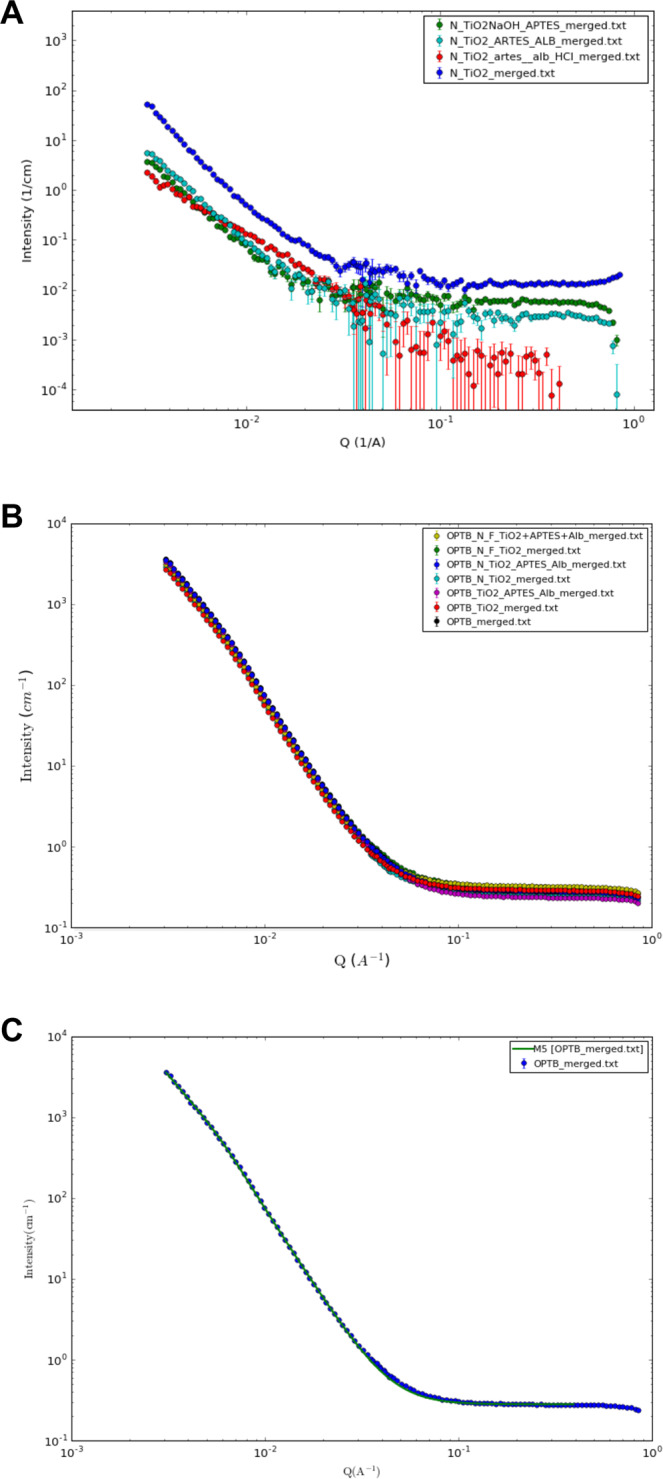
Results from the small-angle neutron scattering of (**A**) N_TiO_2_ (as-synthesized or surface-modified) suspended in D_2_O or D_2_O containing 0.1 M HCl, (**B**) unaltered and experimental dental adhesive resins containing either as-synthesized or surface-modified n-TiO_2_, N_TiO_2_ and NF_TiO_2_ and (**C**) Representative Guinier-Porod fitting unaltered OPTB.

Figure 5(A–C) shows the SANS results for (A) N_TiO_2_ (as-synthesized or surface modified) suspended in D_2_O (with or without HCl [0.1 M]) or (B) dental adhesive resins (unaltered or experimental) containing 20% of nanoparticles (as-synthesized or surface-modified). The results reported in Fig. 5A indicate that, for small values of q (Å^−1^), nanoparticles investigated could be rank ordered in terms of their sizes and agglomeration levels, as follows: N_TiO_2_ > DN_TiO_2_ (NaOH+APTES + Alb)> DN_TiO_2_ (NaOH+APTES) > DN_TiO_2_ (NaOH+APTES + Alb in HCl [0.1 M]). Figure 5B illustrate SANS results for OPTB and experimental dental adhesive resins containing 20% (v/v) of either as-synthesized or surface-modified nanoparticles. Figure 5C illustrates the Guinier-Porod fitting of OPTB. Table 1 illustrates the results for all dental adhesive resins analyzed with SANS. The results in Table 1 were used to determine the morphology, size (*s*), radius of gyration (*Rg*) of scattering objects and the types of interfaces established between nanoparticles and polymeric chains (Porod exponential). It is possible to observe that radius of gyration (in terms of Å) and thickness (in nm) of polymeric chains ranged from 134.99 (N_TiO_2_) to 145.16 (Dn-TiO_2_), and from 46.7 (N_TiO_2_) to 50.2_2_ (Dn-TiO_2_), respectively. The results of the *s* parameter and the Porod exponential (which indicates fractal surface, Table 1) demonstrated the presence of small-sized aggregates (15–50 nm) displaying platelet structures and the establishment of a smooth interface between nanoparticles and the polymeric chains, which indicates the establishment of covalent functionalization of nanoparticles in OPTB.

**Table 1:** Small Angle Neutron Scattering results illustrating the radius of gyration, polymeric chain thicknesses and Porod exponentials for unaltered and experimental dental adhesive resins containing 20% (v/v) of nanoparticles (either doped or co-doped; as-synthesized or surface-modified).

Groups	Radius of Gyration (Å)	Thickness (nm)	Porod Exponential	Background	Scale Factor
OPTB	142.05	49.2	3.7	0.28	0.039
TiO_2_	139.37	48.3	3.7	0.29	0.025
TiO_2__APTES_ALB	145.16	50.2	3.7	0.24	0.042
N_TiO_2_	134.99	46.7	3.7	0.25	0.022
N_TiO_2__APTES_ALB	140.40	48.6	3.7	0.28	0.035
NF_TiO_2_	143.20	49.5	3.7	0.32	0.039
NF_TiO_2__APTES_ALB	136.9	47.1	3.7	0.32	0.025

## Discussion

Approaches to develop second-generation visible light-responsive TiO_2_ nano-catalysts using lanthanide metals (La^3+^, Eu^3+^, ND^3+^, Ce^4+^) and high calcination temperatures have been previously described^39–41^. Xu *et al*.^42^ while investigating a simple route for the preparation of co-doped nanoparticles (NEu_TiO_2_) indicated, based on previous scientific evidence^43^, that traditional calcination approaches results in thermally unstable nanoparticles with reduced surface-to-volume ratios and OH-deficient surfaces.

These factors combined may be translated into nanoparticles requiring multiple functionalization steps and are associated with inferior photocatalytic properties.

Asahi *et al*.^44^ demonstrated that doping titania with non-metal elements, such as nitrogen, narrows the band-gap of TiO_2_ by inserting an intermediary *p* state from nitrogen between the oxygen *2p* states, which extends titania’s optical absorption edge into the visible spectrum (400–750 nm). Despite these promising reports, subsequent studies^45,46^ suggested that N_TiO_2_ translation into practical applications would be restricted due to its deficient reactivity and poor quantum yield. In addition to that, Liu *et al*.^45^ indicated that the artificial lacuna created during the doping process, could negatively impact nanoparticles’ long-term photocatalysis stability.

In the present study undoped (n-TiO_2_), doped (N_TiO_2_) and co-doped (NF_TiO_2_) variations of nanoparticles were synthesized using robust and highly controllable solvothermal reactions. According to Huo *et al*.^27^, this synthesis route yields pure and crystalline TiO_2_ (anatase phase) displaying high levels of nitrogen-doping^47^ in the TiO_2_ network (N/Ti molar ratio = 3.4%) when compared to traditional calcination strategies (N/Ti molar ratio = 1.3%). The results reported by Huo *et al*.^27^ have indicated that nanoparticles fabricated using solvothermal reactions are in fact electron deficient, facilitate the production of electron-hole pairs (by photoinduced processes), display enhanced absorbance of visible light^26^, and consequently, are capable of generating substantial amounts of reactive oxygen species.

According to Bidlack *et al*.^48^ HIM can be used for the characterization of non-sputter-coated biological samples (soft and insulating) with nanometric resolution and outstanding depth of field. In the present study, HIM was used to characterize the surfaces of unaltered and experimental dental adhesive resins. Results reported in the present study have indicated that experimental materials displayed surface characteristics that were comparable to those of OPTB. In addition, phase separation between nanoparticles and the polymeric matrix was not be observed for all nanoparticles and experimental materials investigated. According to Nikolaidis *et al*.^49^ the absence of phase separation in nanofilled dental biomaterials is a good indication of the successful functionalization of nanoparticles into current polymer compositions, and typically translates into experimental materials with superior physical, mechanical and biological properties.

Wang *et al*.^50^ demonstrated that manual incorporation of 10% (wt./wt.) methacryl isobutyl polyhedral oligomeric silsesquioxanes nanoparticles (MI-POSS) resulted in phase separation between nanoparticles and the polymer matrix, and resulted in experimental materials with inferior surface and mechanical properties^50^. According to Abedin *et al*.^51^ phase separation in dental adhesive resins undermines the integrity and durability of the adhesive interface, leads to the leaching of large amounts of unreacted monomers, and may increase the hydrolytic degradation of current polymer compositions.

Small-angle X-ray scattering is a non-destructive, powerful and well-established technique in the field of materials science^52^ that provides averaged structural data over macroscopic sample volumes^53^. Figure 2 illustrates the results from the SAXS experiment for surface-modified N_TiO_2_ suspended in D_2_O (Fig. 2A) or D_2_O containing either NaCl (0.1 M or 1.0 M) or HCl (0.1 M) (Fig. 2B). Figure 2A shows that surface-modified nanoparticles were associated with X-Ray scatterings that were dominated by steep slopes and low intensities (between 4.4937 and 0.0062 a.u.) that varied in a particle-size dependent manner (NaOH <NaOH+APTES < NaOH+APTES + Alb). This finding suggest that each surface modification step resulted in nanoparticles of slightly larger diameters. In addition, X-Ray scatterings suggested that surface-modified nanoparticles tend to agglomerate more when compared to as-synthesized nanoparticles. The results of the present study are in agreement with those reported by Szczerba *et al*.^54^ who demonstrated that X-ray scattering intensity strongly and positively correlates with nanoparticles’ dimensions.

According to Garcia *et al*.^55^ the utilization of small-sized particles (1–20 nm) promotes the formation of nanoparticle agglomerates. Ashraf *et al*.^56^ while investigating the effects of particle size and agglomeration on the properties of nanocomposites have demonstrated that the presence of nano-agglomerates in polymers result in materials displaying low interfacial/interphase properties and poor tensile strength. In a recent study, Garcia *et al*.^57^ have demonstrated the ability of imidazolium ionic solutions (1-n-butyl-3-methylimidazolium tetrafluoroborate) to stabilize the agglomeration of titania quantum dots (size distribution 1.19 nm to 7.11 nm) and resulted in experimental dental adhesive resins displaying promising properties (antibacterial, degree of conversion and adhesion). Pfeiffer *et al*.^58^ while extensively reviewing the literature regarding the impact of ionic environments on the physico-chemical properties of different types of nanoparticles, indicated that pH directly influenced the colloidal stability of ZnO^59,60^, TiO_2_^61^ and AL_2_O_3_^62^ nanoparticles by changing their surface redox potential and electronic stability^63^.

In the present study, Fig. 2B clearly shows that ionic solutions containing NaCl (0.1 M or 1.0 M) or HCl (0.1 M) were capable of modifying the agglomeration behavior of surface-modified nanoparticles, as denoted by X-Ray scatterings dominated by gradual inclines and very high X-ray scattering intensities (between 91.2020 and 0.1744 a.u.). These results indicate that ionic solutions investigated were able to overcome potential negative effects derived from the surface modification strategies investigated. The control over nanoparticles’ agglomeration behavior is anticipated to result in experimental materials displaying superior physical, mechanical and biological properties.

Figure 3(A,B) illustrate the results from ToF-SIMS chemical mapping of specimens fabricated with either unaltered or experimental dental adhesive resins containing 20% (v/v) of nanoparticles (as-synthesized or surface-modified). It is possible to observe that experimental materials displayed ionic fragmentation behaviors that were identical to those observed for OPTB. These findings suggest that the incorporation of nanoparticles did not disturb the molecular makeup of OPTB and seemed to be compatible with the polymeric matrix of the parental polymer. The results presented in Fig. 4(A–D) shows the results from the 2-D ToF-SIMS chemical imaging demonstrating the distribution of Ti^+^ cations on OPTB (4 A) or experimental adhesives containing as-synthesized nanoparticles (n-TiO_2_ [4B], N_TiO_2_ [4 C] and NF_TiO_2_ [4D]). It is clear that the highest amounts of Ti^+^ were observed in Fig. 4C (N_TiO_2_). Kim *et al*.^64^ while extensively reviewing the utilization of ToF-SIMS to probe metal nanoparticles (gold, magnetic and semi-conducting) have indicated that ToF-SIMS can probe the functionalization and location of gold nanoparticles in biological systems, thereby corroborating the findings of the present study regarding the distribution of incorporated nanoparticles.

According to Willumeit^65^ SANS is an accurate and time-resolved instrument with resolutions at the nanometer and subnanometer levels and, therefore, is considered as a powerful tool to investigate the properties of complex materials containing hydrogen. Figure 5A illustrate SANS results for N_TiO_2_ (as-synthesized or surface-modified) suspended in D_2_O or D_2_O + HCl (0.1 M). The findings reported are in agreement with the results from the SAXS experiment, and have indicated that surface-modification strategies used in the present study were indeed successful in grafting APTES and Alb onto the surfaces of metaloxide nanoparticles. In addition, these results corroborate the utilization of low-strength HCl to control nanoparticles’ agglomeration prior to their incorporation and functionalization in experimental dental adhesives. SANS results demonstrate that all materials investigated displayed neutron scattering behavior that were very similar, which indicates that the incorporation of 20% (v/v) of nanoparticles (either as-synthesized or surface-modified) did not adversely impacted the morphology or the structure of polymeric chains in OPTB (in scales from 200-10 nm, correspondent to *q* ranges between 0.003 and 0.1 Å^−1^). These findings have further corroborated the results from HIM and ToF-SIMS regarding the functionalization of nanoparticles in OPTB.

## Conclusions

The present study has successfully demonstrated the synthesis (n-TiO_2_), doping (N_TiO_2_ or NF_TiO_2_) and surface modification (Dn-TiO_2_, DN_TiO_2_, DNF_TiO_2_) of titanium dioxide nanoparticles, as well as, their incorporation into a commercially available dental adhesive resin (OPTB). The present study represents an effort to comprehensively characterize nanoparticles and experimental materials using advanced scientific methodologies, such as Helium-ion microscopy, time-of-flight secondary ions spectrometry and small-angle X-ray and neutron scattering. The present study has shown that surface-modification strategies results in nanoparticles that are larger in diameter and tend to display higher agglomeration levels when compared to as-synthesized N_TiO_2_. The SAXS and SANS results reported have clearly indicated that low-strength ionic solutions may be used to improve the dispersion of nanoparticles prior to their incorporation into dental adhesive resins. The present study has also demonstrated that the incorporation of nanoparticles (undoped or doped; as-synthesize or surface-modified) did not altered the 3-dimensional lamellar distribution of polymer chains and resulted in experimental materials that did not phase separated. SANS results indicated the establishment of smooth interfaces between discrete dispersed nanoparticles and the polymeric matrix, thereby suggesting the attainment of covalent functionalization of nanoparticles in OPTB. It is anticipated that the present study can positively impact the field of dental materials science by offering important information for the development of multifunctional nanofilled materials with promising antibacterial, bioactive and bond-promoting properties. Further optimization and functionalization of nanoparticles in polymer-based dental biomaterials are made necessary to produce the state-of-the-art stimuli-responsive polymers that will be capable of preventing the occurrence of secondary caries.
